# A Rare Case of Cytomegalovirus Retinitis in a Young Immunocompetent Patient

**DOI:** 10.7759/cureus.44948

**Published:** 2023-09-09

**Authors:** Ruchi Shukla, Ashutosh K Mishra, Archana Verma, Pragati Garg, Shrinkhal LNU

**Affiliations:** 1 Department of Ophthalmology, All India Institute of Medical Sciences, Rae Bareli, Rae Bareli, IND; 2 Department of Neurology, All India Institute of Medical Sciences, Rae Bareli, Rae Bareli, IND

**Keywords:** fundus, ganciclovir, immunocompetent, retinitis, cytomegalovirus

## Abstract

Cytomegalovirus (CMV) is known to be the most common opportunistic infection in immunocompromised cases, and CMV retinitis is the most common ocular manifestation. Severe retinitis with involvement of the macula or retinal necrosis can lead to vision loss. Prompt diagnosis and treatment can restrict the disease's progression. We describe the case of a 30-year-old man who presented with the chief complaint of progressive diminution of vision in both eyes for 15 days. Diminution of vision was associated with fever and skin rashes. The patient had no history of diabetes, hypertension, tuberculosis, ocular trauma, ocular surgery, organ transplant history, history of immunosuppression, or previous drug history except paracetamol tablets for fever. On ocular examination on the day of presentation, the patient's best corrected visual acuity on Snellen’s visual acuity chart was 6/12 and 6/24 in the right and left eyes, respectively. Fundus examination revealed a well-defined optic disc with peripapillary flame-shaped hemorrhages with exudates and an epiretinal membrane. On spectral domain optical coherence tomography (SD-OCT), macular edema was 469 µm and 421 µm in the right and left eyes, respectively. On serological examination, only cytomegalovirus IgG came out positive (1196.65 AU/ml). Based on the clinical findings, fundus examination, and lab investigations, the patient was diagnosed as having a systemic CMV infection with CMV retinitis, and treatment was started with intravenous ganciclovir. With timely diagnosis and management, the patient’s vision was recovered. This is a rare case report regarding the development of CMV retinitis in a completely immunocompetent individual.

## Introduction

The cytomegalovirus (CMV) virus belongs to the *Herpesviridae* family. Cytomegalovirus retinitis (CMVR) is due to the hematogenous spread of CMV. CMV retinitis is the most common opportunistic ocular infection in immunocompromised patients, particularly in acquired immunodeficiency syndrome (AIDS) patients where there is a low CD4 count [1].

CMVR is a clinical indicator of immune deficiency and occurs late in the course of the disease. The symptoms of CMV retinitis depend on the location of the lesions in the retina. CMV retinitis threatens the vision of most immunosuppressed individuals, but very few cases have been reported in healthy individuals [2]. Here, we report a case of bilateral CMV retinitis in a fully immunocompetent, healthy individual without any predisposing risk factor for CMV retinitis.

## Case presentation

A 30-year-old man was referred to the outpatient department of ophthalmology from the department of neurology with the chief complaint of diminution of vision in both eyes for 15 days.

The patient was apparently asymptomatic 15 days ago when he developed a high-grade fever, which was gradual in onset and was relieved on medications. The patient also complained of headaches and lower limb pain, which increased when performing any activity and as the day progressed. Fever was also associated with painless skin rashes that had a flat, red area with small confluent bumps. Skin rashes started at the neck 15 days ago, gradually progressing to the left side of the face and eyelids and later on to the right side.

Diminution of vision was painless, insidious in onset, and gradually progressive to the present status. Diminution of vision was greater in the left eye. It was associated with flashes, periorbital pain, headaches, and heaviness in the left eye. There was no history of diplopia or floaters.

There was no history of diabetes, hypertension, tuberculosis, ocular trauma, ocular surgery, organ transplant history, history of immunosuppression, or previous drug history except paracetamol tablets for fever.

On the day of presentation, the patient was conscious and oriented; his Glasgow Coma Scale (GCS) was E4V5M6, and he was afebrile.

A dermatology reference was taken for skin rashes. Skin examination revealed erythematous maculopapular rashes over the face, neck, and legs, and a diagnosis of viral exanthema was made.

**Figure 1:**
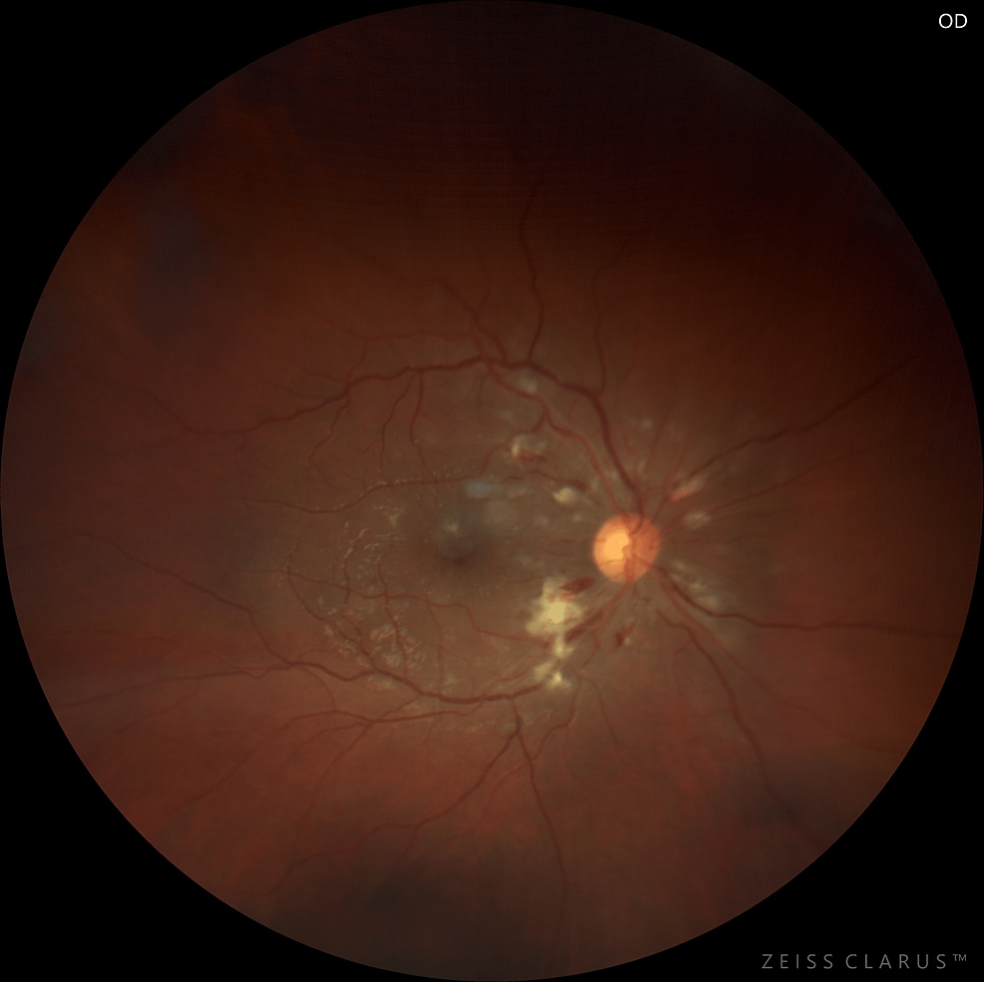
Fundus photograph of the right eye on the day of admission

**Figure 2:**
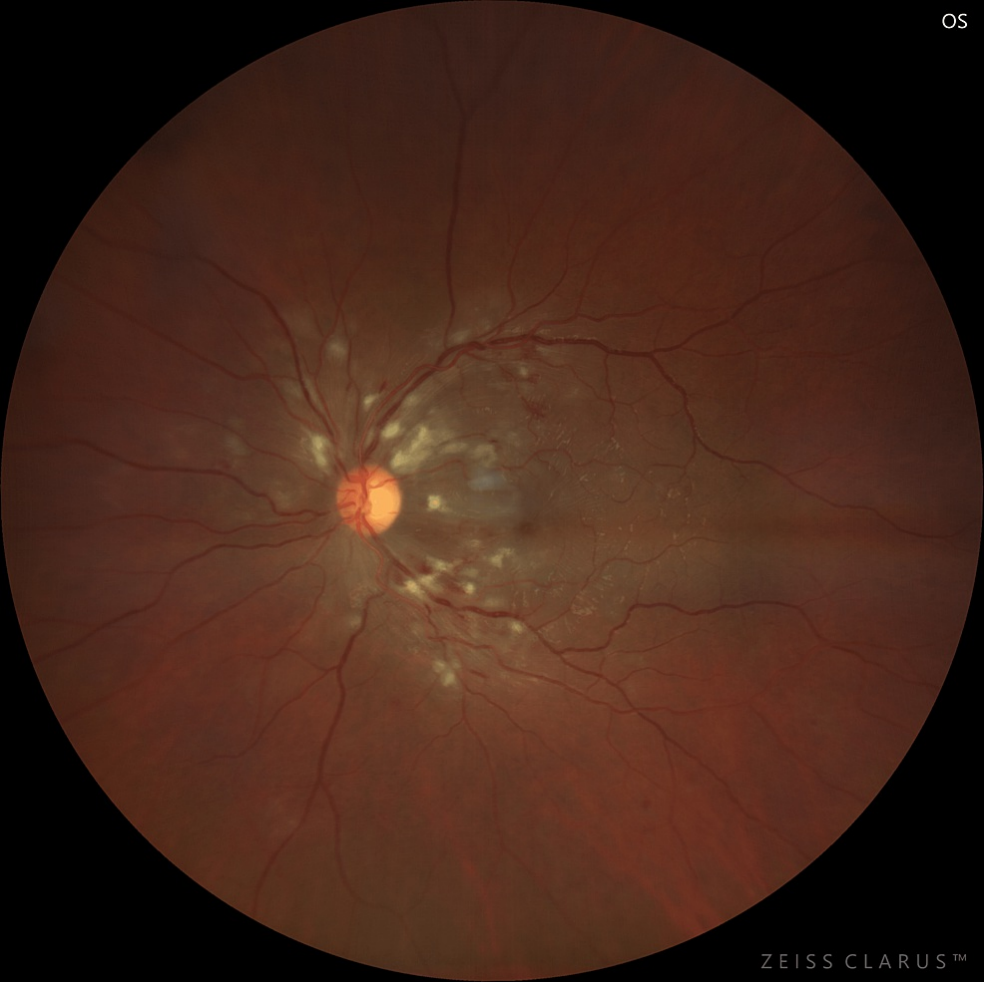
Fundus photograph of the left eye on the day of admission

**Figure 3:**
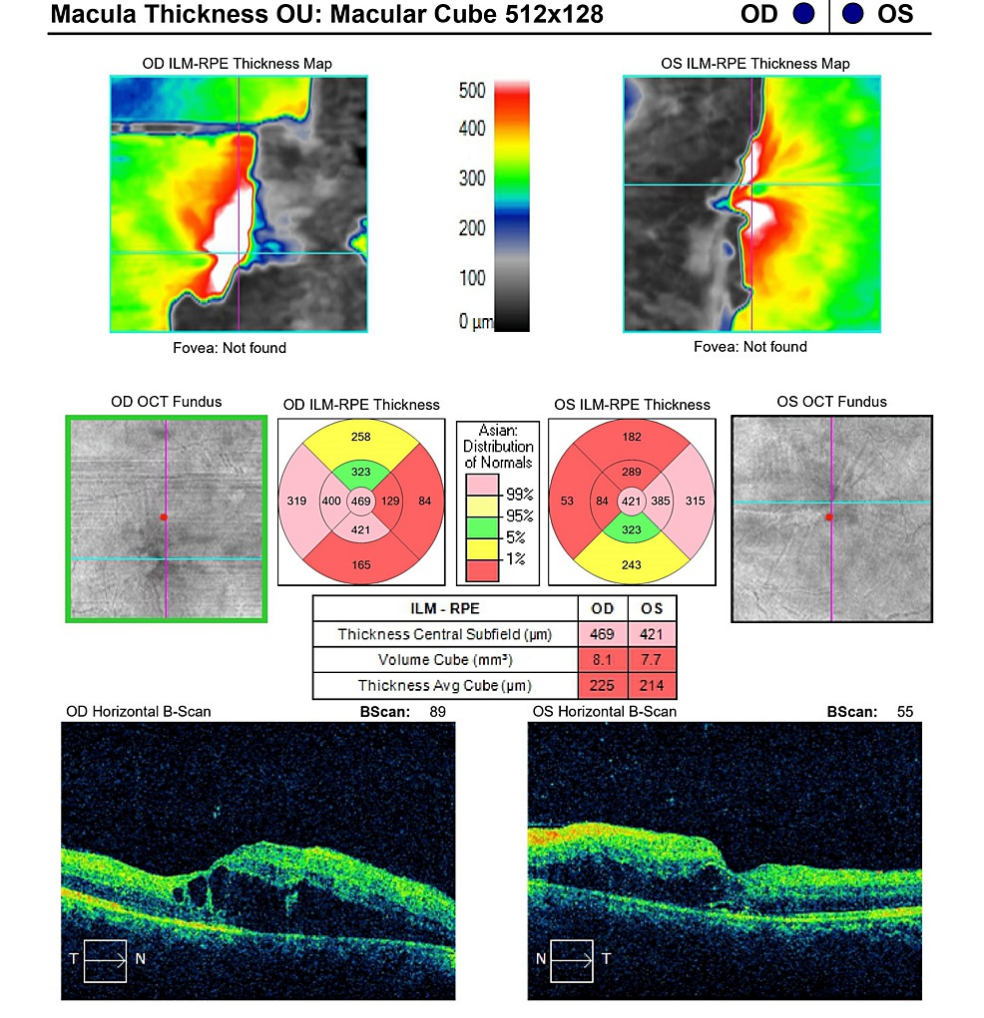
Optical coherence tomography of the macula of both eyes on the day of admission

On ocular examination on the day of presentation, the patient's best corrected visual acuity on Snellen’s visual acuity chart was 6/24 and 6/36 in the right and left eyes, respectively. On slit lamp examination, the anterior segment was quiet and was within normal limits. The pupils were of normal size and were reacting normally. The intraocular pressure was 15 mmHg in both eyes, respectively. Fundus examination of both eyes revealed a well-defined optic disc with peripapillary flame-shaped hemorrhages with exudates and an epiretinal membrane (Figures 1, 2). On spectral domain optical coherence tomography (SD-OCT), macular oedema was 469 µm and 421 µm in the right and left eyes, respectively (Figure 3).

Due to the unusual presentation and atypical findings on the fundus, multiple infectious and hematological workups were planned. The patient underwent a complete blood count, erythrocyte sedimentation rate, blood sugar, dengue rapid test, liver function, renal function, creatinine kinase, venereal disease research laboratory test, rheumatoid factor, anti-converting enzyme, antinuclear antibodies, chest X-ray, and Monteux test. The patient’s serum was tested for viral markers such as hepatitis C antibody test, HIV antibody test, hepatitis B viral antigen test, TORCH test (immunoassay of toxoplasma, rubella, cytomegalovirus, and herpes simplex virus), and CD4 count. Out of this enormous number of tests, only cytomegalovirus IgG came out positive (1196.65 AU/ml). The patient denied consent for a vitreous fluid biopsy for PCR testing. To exclude any underlying immunodeficiency or malignancy, an autoimmune panel was performed, and a physician consultation was taken, but all the workups came out negative.

The patient had undergone a computed tomography scan of the brain elsewhere 12 days ago, which revealed a calcific granuloma in the right occipital lobe, whereas magnetic resonance imaging of the brain and orbit on the day of presentation did not show any abnormality.

Based on the clinical findings, fundus examination, and lab investigations, the patient was diagnosed as having a systemic CMV infection with CMV retinitis. Based on our diagnosis, treatment was started with intravenous ganciclovir. An induction dose of 5 mg/kg (over 1 hour) every 12 hours for 14 days was given, followed by a maintenance dose of 5 mg/kg daily for seven days/week for one week thereafter, and then the patient was shifted to oral ganciclovir 1000mg six times/day. Considering the hematological toxicity and effect on renal function of intravenous ganciclovir, a complete blood count and renal function test were performed on each day of the dose. Frequent ophthalmological examinations were performed to assess the effect of treatment on the fundus findings.

Evaluating the fundus findings one month from the day of presentation, there was resorption of retinal hemorrhages with significant resolution of foveal edema, with some leftover edema in the perifoveal area. On SD-OCT, the central macular thickness (CMT) was 244 µm and 265 µm in the right and left eyes, respectively. During the course of the treatment, the patient's vision improved on each visit, and the patient was on follow-up for six months. At the six-month visit, vision improved to 6/6 on Snellen’s visual acuity chart in both eyes, respectively.

**Figure 4:**
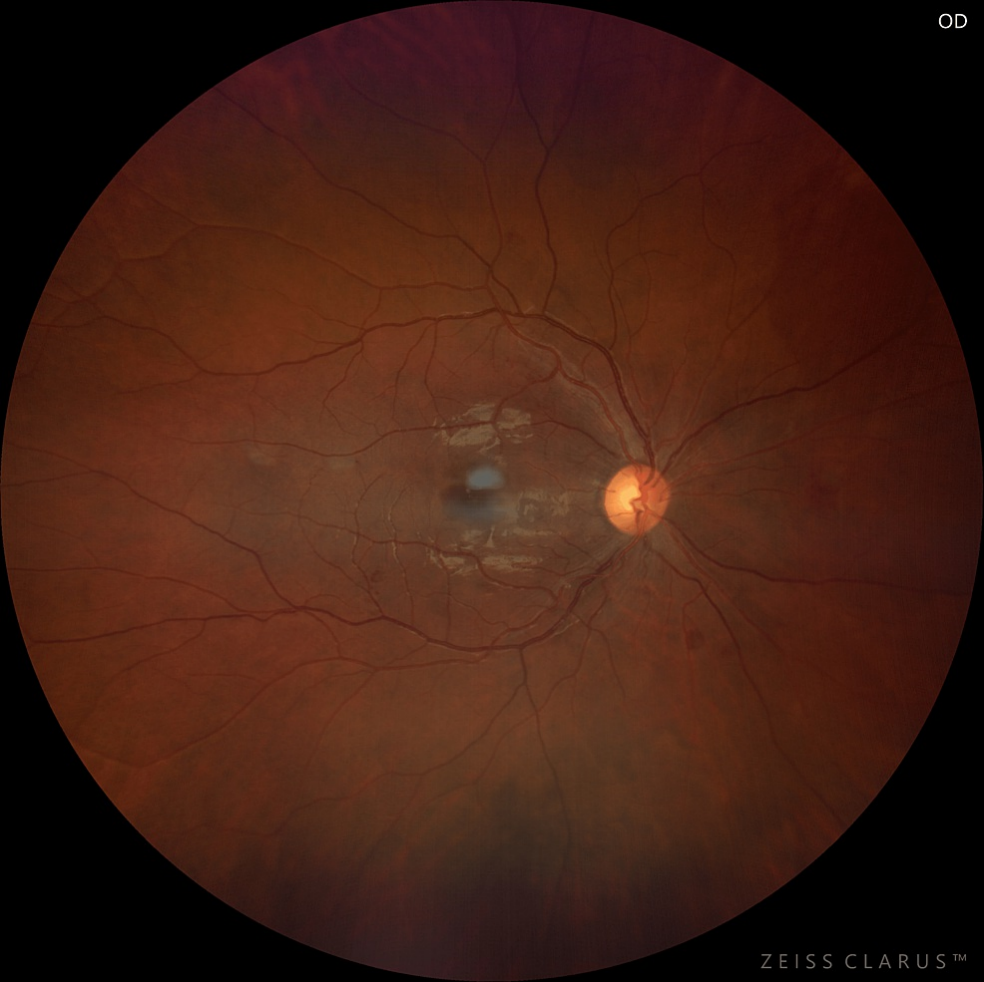
Fundus photograph of the right eye at six-month follow-up

**Figure 5:**
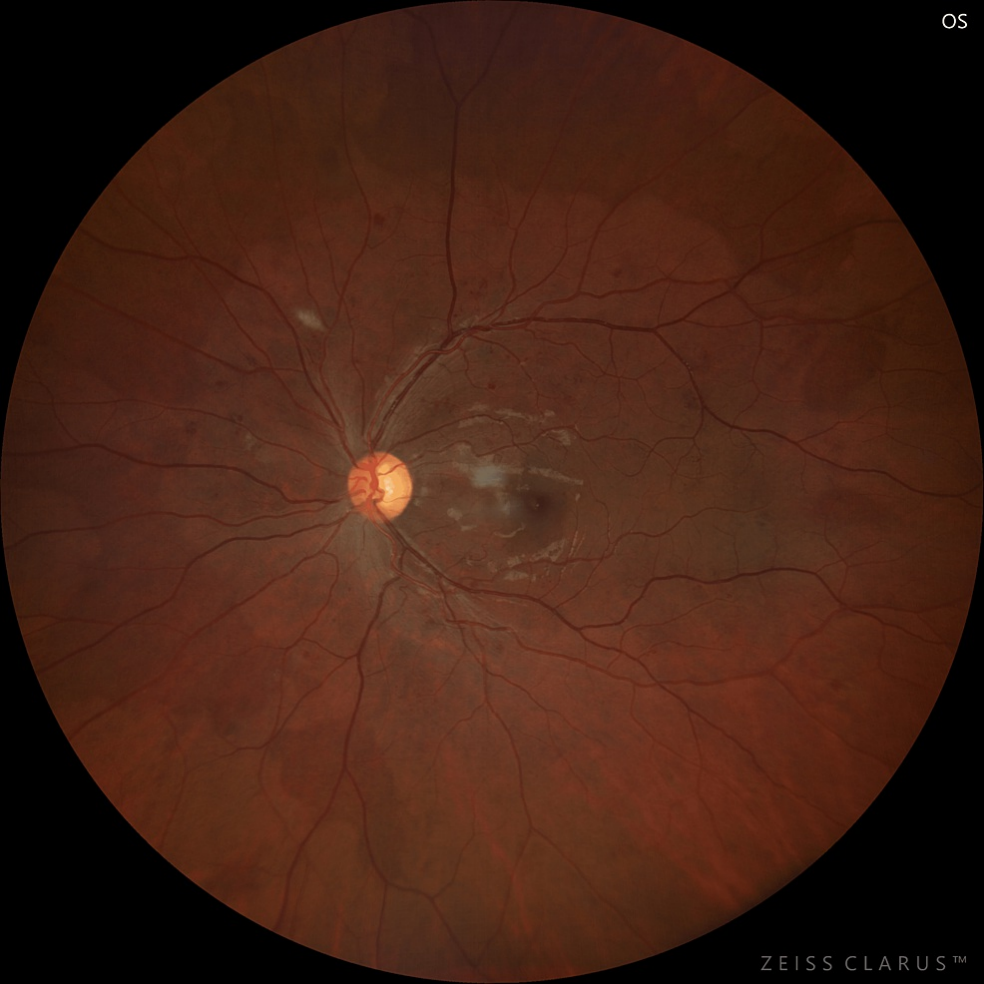
Fundus photograph of the left eye at six-month follow-up

**Figure 6:**
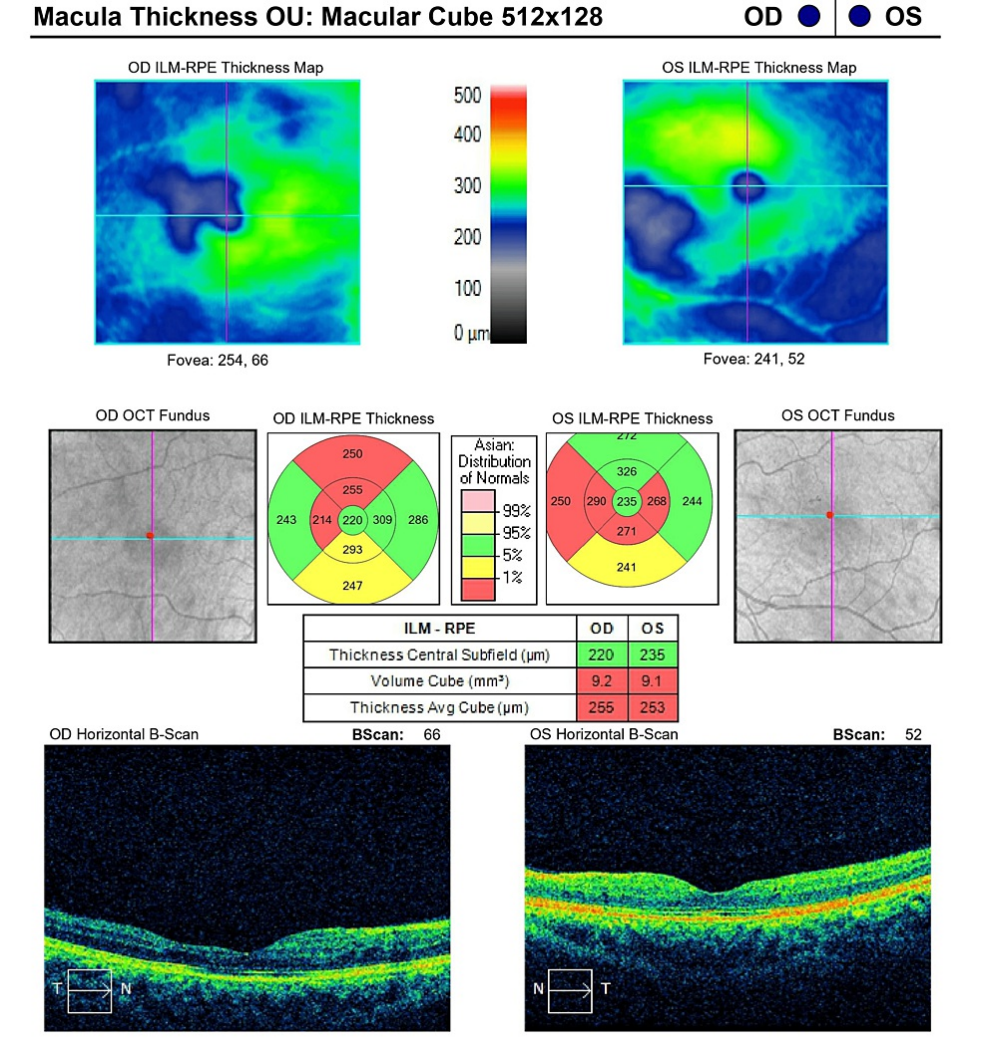
Optical coherence tomography of the macula of both eyes at six-month follow-up

At six months, on fundus examination of both eyes, a near total resolution of macular oedema was seen with only a few scattered dot and blot hemorrhages (Figures 4, 5). On SD-OCT, CMT was 220 µm and 235 µm in the right and left eyes, respectively (Figure 6).

## Discussion

CMV infection is one of the most common viral infections in the world and spreads through a hematogenous route to the retina, infecting the vascular endothelium and the retinal cells to cause CMV retinitis [3]. CMV has varied ocular presentations, which include keratic precipitates, anterior chamber reactions, retinitis, vasculitis, and vitritis. CMV retinitis is a common opportunistic infection that mainly affects individuals in immunocompromised states. CMV retinitis has been well studied in immunocompromised conditions such as patients suffering from AIDS, individuals on immunosuppressants, organ transplantation patients, malignancies, and any condition that leads to a decline in immune function [2]. This can lead to CMV reactivation and cause CMV retinitis. In its severe form, CMV retinitis can lead to vision loss.

Patients with non-immunological conditions that cause limited immune dysfunction, such as diabetes, advanced age, hypertension, and intravitreal injections, can also acquire CMV retinitis if the virus is able to cross the blood-retinal barrier [4,5].

It is not common for immunocompetent individuals to encounter CMV retinitis, but a few cases have been reported in the literature. The majority of case reports reported were related to management with intravitreal injections of corticosteroids, and the cause postulated behind this can be due to the reactivation of CMV-infected leucocytes, which leads to local immunosuppression [6-8].

A few case reports of CMV retinitis in immunocompetent individuals had diabetes and were in the elderly age group, which are both risk factors for acquiring CMV infection. Several mechanisms have been suggested regarding the alteration in the immune system in cases of diabetes, such as a reduction in cytokines and impaired phagocytosis. Infected leucocytes get entrapped in the retina and cause CMV retinitis in an immunocompetent individual with diabetes. A similar mechanism has been said to work in hypercoagulable states like hypertension [9,10].

On a thorough review of the literature, only two case reports that documented CMV retinitis in previously healthy individuals who were young female adults were found, but both of them were published in the 1970s and 1980s, when there was no availability of proper investigations and treatment [11,12].

Although patients who are in immunocompromised states such as AIDS, organ transplantation, and lymphoma and those in immunosuppressed conditions such as diabetes or local immunosuppression have an increased risk of developing CMV retinitis, healthy individuals with no such immunosuppression can acquire CMV infection and manifest it as CMV retinitis.

The explanation for a healthy person developing CMV retinitis remains unclear despite an array of investigations. It can be hypothesized that there may be a genetic susceptibility that acts as a predisposing factor. However, further research is needed to study the cause of CMV retinitis in completely immunocompetent individuals.

There has been a debate regarding the use of antiviral therapy in immunocompetent patients with CMV infection. In a study by Yoshinaga, CMV retinitis resolved without antiviral therapy, but some physicians are in favor of giving appropriate antiviral therapy, especially when there is ocular involvement [13]. Intravenous ganciclovir is efficacious in treating CMV retinitis despite having some side effects. It has the added advantage of restricting systemic dissemination compared to intravitreal injections [14]. In our case, with timely diagnosis and management, the patient’s vision was recovered.

In immunocompetent patients, primary CMV infection presents as a viral syndrome, and the manifestations are fever and myalgia; hence, a thorough and comprehensive evaluation is needed to rule out other causes of fever. Physicians and ophthalmologists should consider CMV infection in their differential diagnosis of patients with fever and malaise with retinal findings, even if they are immunocompetent.

CMV should also be considered a differential diagnosis in all cases of retinitis, along with herpes simplex virus, varicella zoster virus, toxoplasmosis, and syphilis.

## Conclusions

As CMV is a treatable infection, the course of CMV retinitis can be controlled with quick diagnosis and treatment. CMV is known to cause a variety of systemic and ocular symptoms in immunocompromised individuals. This case report highlights the possibility of CMV retinitis in a person without a known immunocompromised state. Therefore, the patient's immunological status shouldn't be viewed as a roadblock to suspecting and then diagnosing CMV retinitis.
